# Robotic-assisted total gastrectomy for refractory hypoproteinemia in Menetrier’s disease: a case report with operative video and literature review

**DOI:** 10.3389/fmed.2025.1634451

**Published:** 2025-09-17

**Authors:** Lang Wang, Ziping Liu, Jing Zhang, Xianglin Zhu, Shijun Zhao, Cheng Zhao, Hao Liang, Jie Zhang, Tian Gao, Yinlu Ding

**Affiliations:** Department of Gastrointestinal Surgery, The Second Qilu Hospital of Shandong University, Jinan, China

**Keywords:** Ménétrier’s disease, robotic-assisted total gastrectomy, refractory hypoproteinemia, Roux-en-Y esophagojejunostomy, case report

## Abstract

Ménétrier’s disease is a rare, progressive disorder of unclear etiology, typically affecting middle-aged men and characterized by giant gastric mucosal folds, mainly in the fundus and body, with occasional antral involvement. Diagnosis is challenging due to its rarity and the need to differentiate from hypertrophic lymphocytic gastritis, Zollinger-Ellison syndrome, gastric cancer, and lymphoma. We report a young male with Menetrier’s disease presenting as generalized edema due to hypoproteinemia. After a challenging diagnostic process and ineffective medical management, the patient underwent robot-assisted total gastrectomy with Roux-en-Y esophagojejunostomy, which corrected the hypoproteinemia. He recovered uneventfully and was discharged on postoperative day 12. At 23 months post-surgery, he showed no hypoalbuminemia, edema, or related symptoms; body weight and serum albumin remained normal. Robot-assisted total gastrectomy is a surgical method for treating Meniere’s disease; however, its cost-effectiveness should be carefully considered, and in the long term, more high-quality studies may be required to validate its feasibility.

## 1 Introduction

Ménétrier’s disease, also known as giant hypertrophic gastritis or protein-losing hypertrophic gastropathy, is a rare and severe gastric disorder associated with neoplastic potential (1, 2). Fewer than 1,000 cases have been reported since its initial description in 1888, with a predilection for males aged 30–60 years (3). The precise etiology remains incompletely understood, though it is generally considered an acquired condition. In pediatric cases, Ménétrier’s disease has been linked to cytomegalovirus infection and typically follows a self-limited course (4), whereas in adults, *Helicobacter pylori* infection is implicated (5, 6). Overexpression of transforming growth factor alpha (TGF-*α*) in the gastric epithelium also contributes to pathogenesis (7–9), and SMAD4 mutations have been reported as a potential etiological factor (10). The disease predominantly involves the gastric body and fundus, though antral involvement has also been documented (11–13). Endoscopically, the gastric mucosa exhibits characteristic gyriform thickening (3, 13), while histopathology reveals foveolar hyperplasia, glandular atrophy, and a reduction in acid-secreting parietal cells (14). Common clinical manifestations include epigastric pain, fatigue, anorexia, weight loss, edema, and vomiting (2, 3, 15, 16). Given its progressive nature and classification as a premalignant lesion (17), coupled with diagnostic challenges, a comprehensive understanding of its management is essential. Here, we present a case of Ménétrier’s disease in a young male, characterized by generalized edema secondary to hypoalbuminemia, detailing the complex diagnostic and therapeutic course, and providing insights into the diagnosis and management of this rare entity.

## 2 Case presentation

### 2.1 Complex medical history

A 38-year-old male with a history of external hemorrhoidectomy (4 years prior) and a family history of paternal lung cancer presented with generalized edema, predominantly in the lower extremities, and abdominal distension for over 10 days. Initial workup revealed hypoalbuminemia (albumin 23.4 g/L), hypocalcemia (calcium 1.79 mmol/L), with unremarkable urinalysis, tumor markers, BNP, viral panel, and chest-abdominal CT. Gastrointestinal endoscopy identified lesions in the gastric fundus and body; biopsy showed acute and chronic gastritis, reduced gland density, epithelial hyperplasia, and focal diffuse large cells. Pathology review at a tertiary center revealed chronic mucosal inflammation with focal high-grade intraepithelial neoplasia; immunohistochemistry: Ki-67 (hotspot ~40%), CK(AE1/AE3)(+), CEA(−), P53 (wild-type+). Liver function showed further decreased albumin (17.8 g/L); immunological and rheumatological tests were unremarkable. Symptomatic treatment with albumin infusion led to symptom improvement and discharge.

After discharge, the patient’s symptoms worsened and he was readmitted locally. Tests showed 24-h urinary protein at 0.19 g, albumin at 17.7 g/L, and no abnormalities in rheumatologic markers. Symptomatic treatment with diuretics and albumin improved abdominal distension, but bilateral lower limb edema with erythema, warmth, pain, and fever up to 38.6°C persisted. Ceftriaxone and other therapies yielded no significant improvement.

The patient presented to the nephrology department with eyelid edema and mild pitting edema of both lower limbs. Laboratory evaluation revealed hypoalbuminemia (18.4 g/L), decreased globulin (15.6 g/L), and reduced A/G ratio (1.18); fecal occult blood was positive. Infectious soft tissue disease of the lower limbs was considered the cause of fever. Despite albumin infusion, diuretics, and anti-infective therapy, hypoalbuminemia persisted, though hypocalcemia was corrected after albumin supplementation. Multidisciplinary consultation excluded cardiac, hepatic, and renal dysfunction as causes of systemic edema. Further investigations, including peripheral blood morphology, Brucella, *C. difficile*, tuberculosis, CMV, *H. pylori* testing, and enhanced CT, were performed. Pathology review of gastric biopsies from an outside hospital revealed severe chronic active gastritis with marked erosion, glandular destruction, and prominent regenerative changes; low-grade gastric adenocarcinoma could not be excluded. Immunohistochemistry: CEA (−), CK (+), Ki67 (high in proliferative zone), P53 (wild-type). Repeat endoscopy showed abundant gelatinous exudate, diffuse congestion, edema, and thickened folds in the gastric fundus and body. Large EMR biopsy was performed (Supplementary Figures S1A–E). The chief gastroenterologist considered gastric disease the likely cause of hypoalbuminemia. During hospitalization, lower limb infection improved with antibiotics, and albumin, IVIG, and PPI were administered for hypoalbuminemia-related edema, with partial symptomatic improvement. The patient was discharged while awaiting pathology results.

One week post-discharge, the patient developed generalized edema and abdominal distension, and was readmitted to the Department of Gastroenterology. Gastroscopic biopsy revealed severe chronic active gastritis with erosion, congestion, edema, marked proliferation and elongation of foveolar and glandular neck regions, partial glandular dilatation, significant reduction of oxyntic glands, and focal pyloric gland metaplasia, consistent with Menetrier’s disease based on clinical and endoscopic findings. Immunohistochemistry: CEA (foveolar surface membrane +), CK (broad-spectrum +), Ki67 (normal proliferative zone pattern), MUC-2 (−), MUC-5 AC (foveolar epithelium +), MUC-6 (metaplastic pyloric glands +), P53 (wild-type expression) (Supplementary Figure S1F). During hospitalization, albumin supplementation, acid suppression, and octreotide were administered with poor response. Multidisciplinary discussion concluded the disease is difficult to treat, optimal therapy remains unclear, and prognosis is poor; further evaluation for gastrectomy was recommended. After being informed, the patient chose to discharge voluntarily.

After discharge, the patient visited a higher-level gastroenterology department outside the province. Further rheumatologic, CMV, *H. pylori*, gastrointestinal tumor markers, lymphocyte subsets, and comprehensive humoral immunity tests remained unremarkable. Enhanced retroperitoneal CT showed mild gastric wall thickening, prompting gastroscopy. Gastroscopy revealed diffuse lesions in the gastric fundus and body, circumferentially thickened folds with gyriform changes, surface congestion and erosion, abundant white mucus in the lumen, poor response to chymotrypsin-protease irrigation, marked mucosal friability with contact bleeding, and diffuse submucosal thickening (10–15 mm), highly suggestive of hypertrophic gastritis. Large mucosal and deep gastric biopsies were obtained. Pathology reviewed by multiple gastrointestinal pathologists indicated Menetrier’s disease, with surgical intervention recommended. The patient subsequently self-discharged and presented to our department.

**Figure 1:**
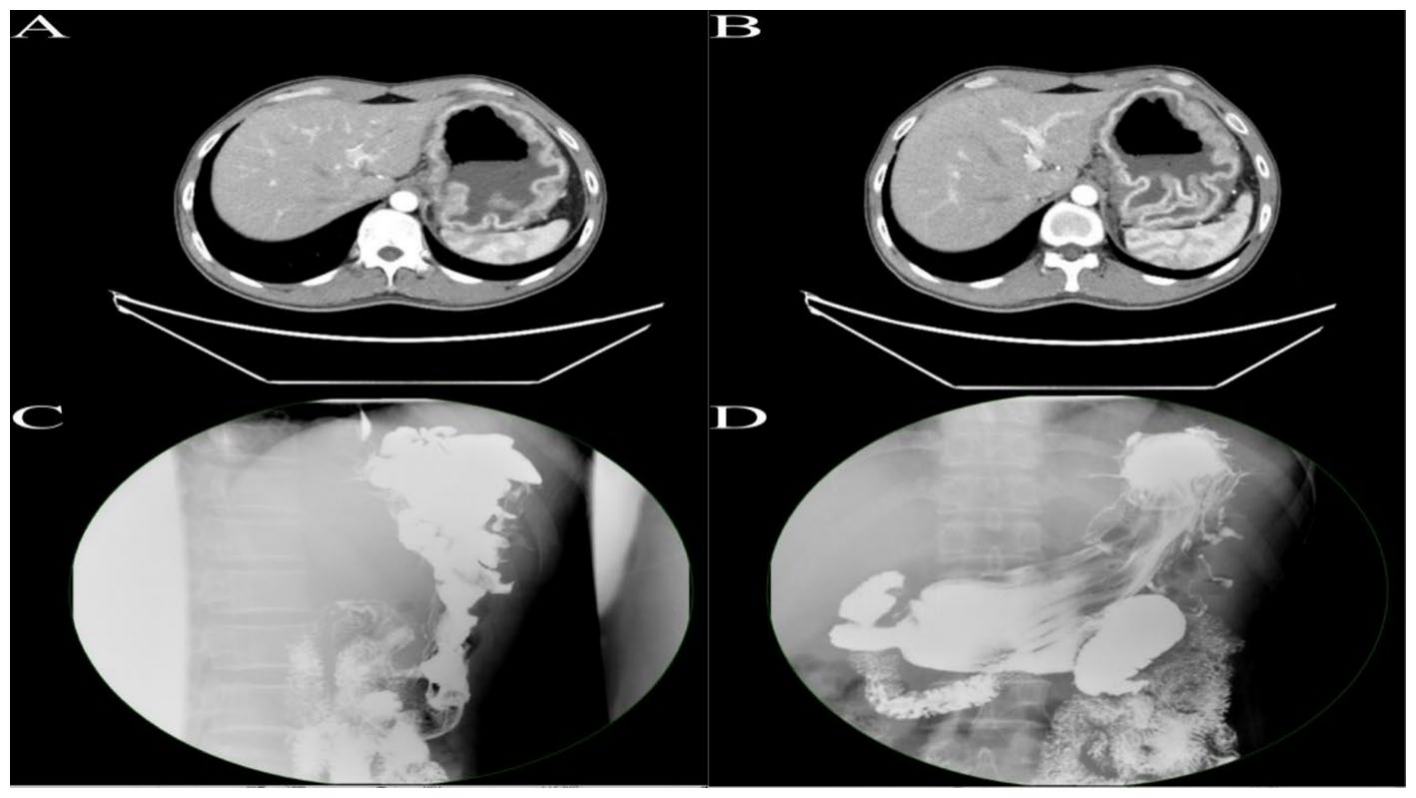
**(A,B)** Enhanced CT of the upper abdomen reveals gastric distension with significant mucosal thickening exhibiting a corrugated appearance, with pronounced enhancement on contrast imaging. **(C,D)** Upper gastrointestinal series demonstrates marked hypertrophy of the mucosal folds in the fundus and body of the stomach, presenting with polypoid and convoluted characteristics, with some interfold spaces appearing irregular.

After admission, upper gastrointestinal contrast study and contrast-enhanced CT of the chest, abdomen, and pelvis confirmed the diagnosis of Menetrier’s disease involving the gastric fundus and body (Figure 1). Multidisciplinary discussion determined the lesion was too extensive for ESD, recommending surgical intervention with total gastrectomy. Following preoperative optimization and exclusion of surgical contraindications, and in accordance with the patient’s wishes, robot-assisted total gastrectomy with Roux-en-Y esophagojejunostomy was planned.

### 2.2 Surgical procedure

**Figure 2:**
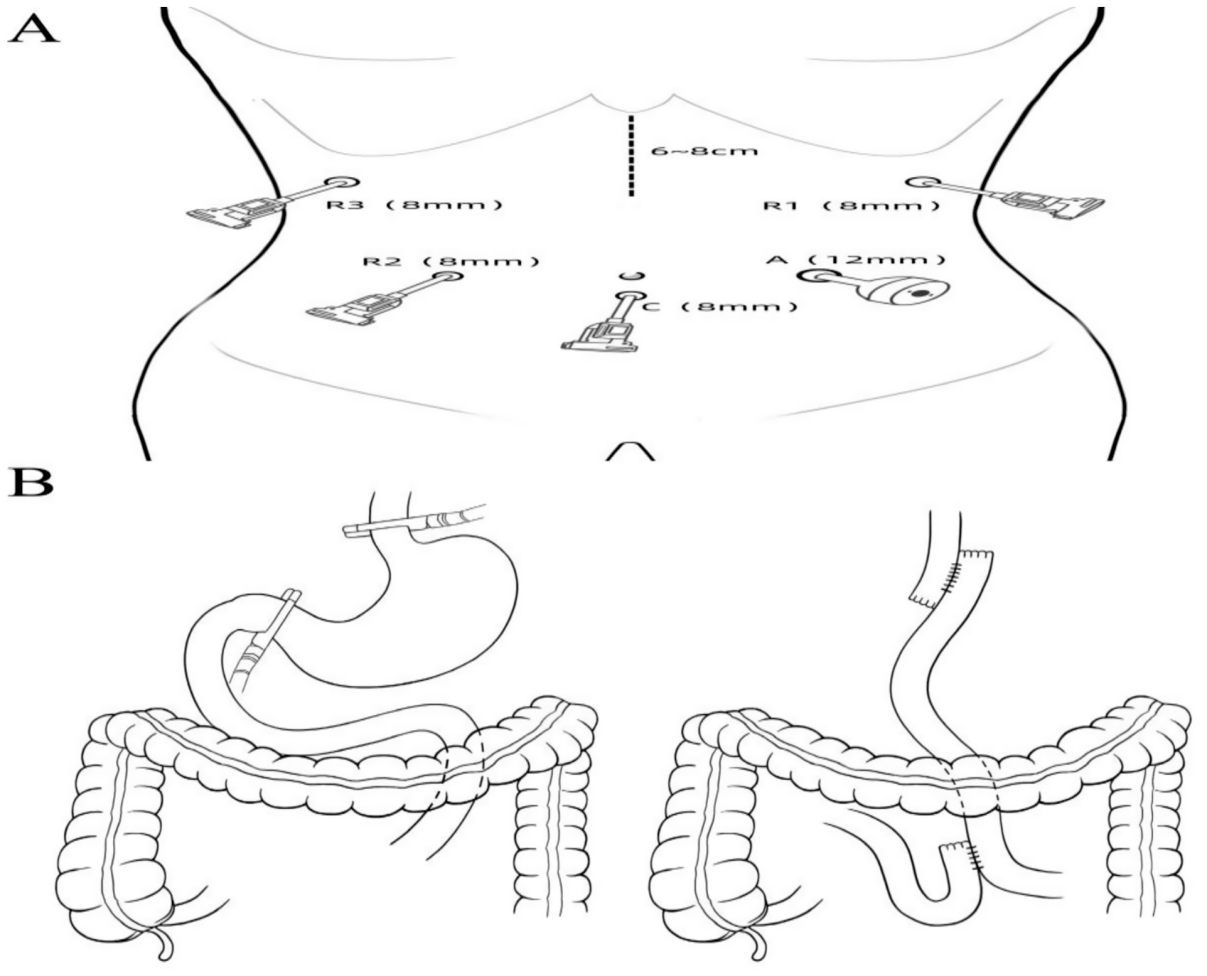
**(A)** Trocar placement for surgical access. **(B)** Schematic illustration of the extent of surgical resection and digestive tract reconstruction.

**Figure 3:**
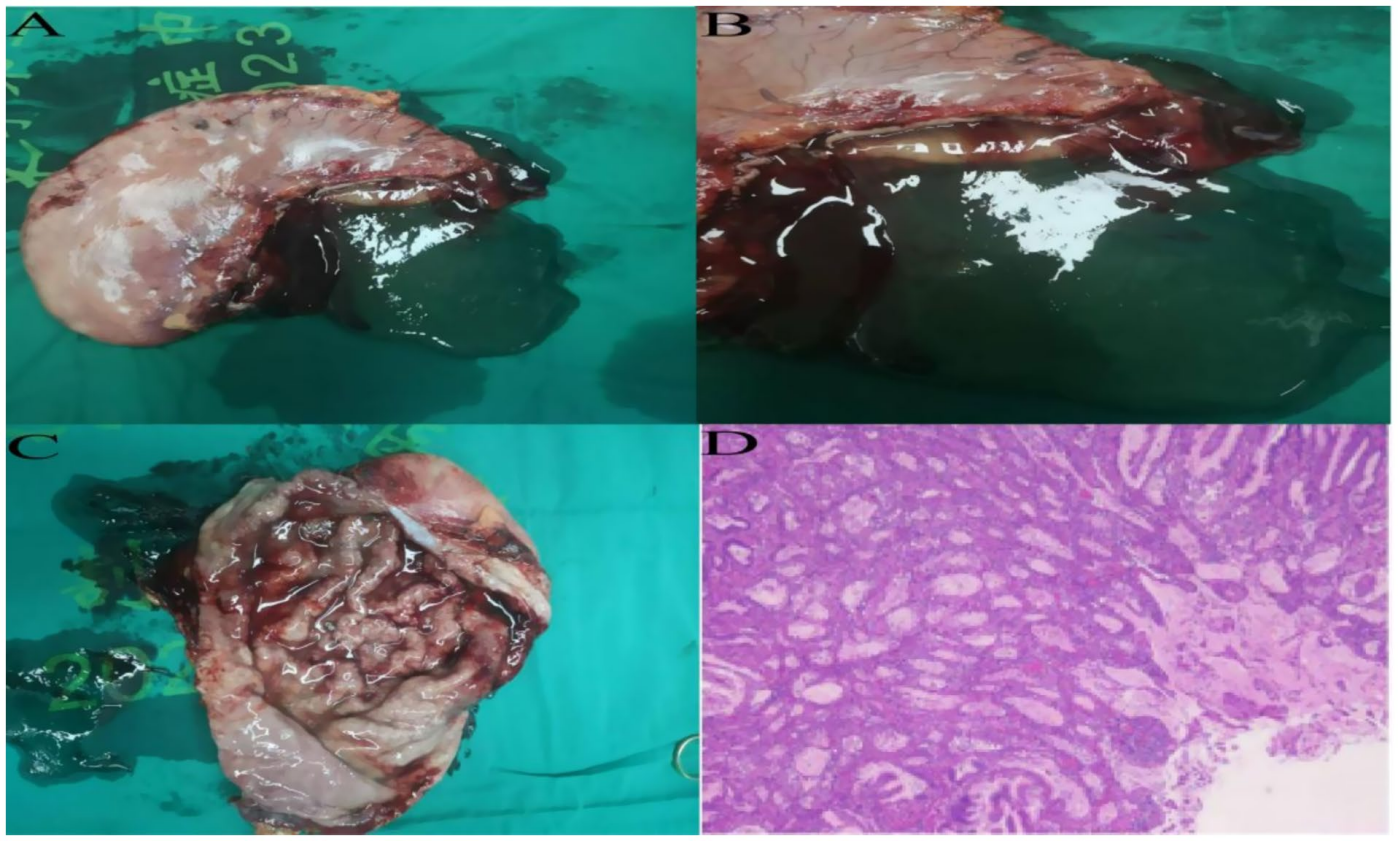
**(A–C)** Postoperative total gastrectomy specimen reveals diffuse thickening of the gastric wall, with prominent and disorganized mucosal folds, and a significant accumulation of yellow-white viscous secretions within the gastric lumen. **(D)** Postoperative specimen routine pathology results, hematoxylin and eosin staining, high-power microscopy.

1. After successful general anesthesia, a gastric tube, urinary catheter, and central venous catheter were placed. The patient was positioned supine with legs apart, and standard sterile draping was performed. A 1 cm infraumbilical incision was made for pneumoperitoneum with CO2 insufflation maintained at 12 mmHg. A trocar was inserted as the camera port (C, 8 mm). The patient was placed in a 15° Trendelenburg position. Exploration assessed the diaphragm, liver, paracolic gutters, pelvis, small intestine, peritoneum, omentum, and mesentery. Findings included diffuse gastric wall thickening and edema, firm texture, significant gastric enlargement, multiple soft perigastric lymph nodes (~5 mm), extensive adhesions, marked perigastric vascular proliferation, and mild pelvic effusion. Robotic-assisted total gastrectomy was deemed feasible. Additional ports were placed: R1 (8 mm, left anterior axillary line, subcostal), for harmonic scalpel; R2 (8 mm, right midclavicular line, umbilical level), for Maryland bipolar forceps; R3 (8 mm, right anterior axillary line, subcostal), for Cadiere forceps; and A (12 mm, left midclavicular line, umbilical level), as an assistant port. The “arc-shaped five-port” configuration was used (Figure 2A). The liver was suspended to fully expose the subphrenic cardia and esophagus.
2. After entering the lesser sac, the omentum is dissected leftward along the gastric wall, ligating and dividing the left gastroepiploic vessels near the lower pole of the spleen. Several short gastric vessels are divided superiorly. The gastric fundus is retracted inferolaterally to expose the left cardia and left crus of the diaphragm. Dissection continues along the greater curvature to the right, exposing the pancreatic head at the lower pancreatic border. The right gastroepiploic vessels are exposed, ligated, and divided at their roots, followed by ligation and division of the inferior pyloric vessels. The duodenal bulb is mobilized up to 2 cm below the pylorus and transected with a 60 mm laparoscopic linear stapler (white cartridge, 2.6 mm staple height). The assistant retracts the gastric stump superolaterally to expose the pancreas, and the pancreatic capsule is dissected to the upper border. The right gastric vessels are exposed, ligated, and divided, then the stomach is further retracted to expose and divide the left gastric vessels. The posterior gastric wall is elevated, and posterior gastric vessels are ligated and divided. The lesser omentum is dissected along the liver margin to the right cardia and right crus. The esophagus is fully mobilized and transected with a 60 mm laparoscopic linear stapler (blue cartridge, 3.6 mm staple height). Both sides of the esophageal stump are suspended with 3–0 absorbable knotless sutures (15 cm) to prevent retraction. A 0.5 cm incision is made in the posterior wall of the esophageal stump with electrocautery, followed by exploration and dilation with dissecting forceps, and a gastric tube is advanced through the incision.
3. A 6–8 cm upper midline incision was made below the xiphoid, with layered entry into the abdomen and placement of a wound protector for specimen retrieval. The jejunum was transected approximately 25 cm distal to the ligament of Treitz using a 60 mm laparoscopic linear stapler (white cartridge, 2.6 mm staple height). The distal jejunum was brought up retrocolically to the esophageal stump without tension. A 0.5 cm mesenteric-side enterotomy was made and dilated at the jejunal limb, 40 cm from the distal end, and at the proximal and distal stumps. Side-to-side jejunojejunostomy was performed with a 60 mm linear stapler (white cartridge, 2.6 mm), and the common enterotomy and stump were closed with 3–0 absorbable knotless sutures, reinforcing the duodenal stump. Mesenteric defects were closed. After re-establishing pneumoperitoneum, an esophagojejunostomy (overlap technique) was performed at the distal jejunal stump, with closure and reinforcement using 3–0 absorbable knotless sutures. Resection and anastomosis diagrams are shown in Figure 2B.
4. The abdominal cavity was irrigated with warm saline and hemostasis achieved. Two drains were placed—one behind the esophagojejunostomy and one in the left upper abdomen—exteriorized and secured through lateral abdominal wall trocar sites (Supplementary Video S1). Instrument and gauze counts were accurate. The abdomen and trocar sites were closed in layers. Gross specimen showed diffusely thickened gastric wall, coarse and disordered mucosal folds, and abundant yellow-white viscous secretions in the gastric lumen (Figures 3A–C). The procedure was uneventful, lasting 245 min with approximately 20 mL intraoperative blood loss.

### 2.3 Postoperative outcome

**Figure 4:**
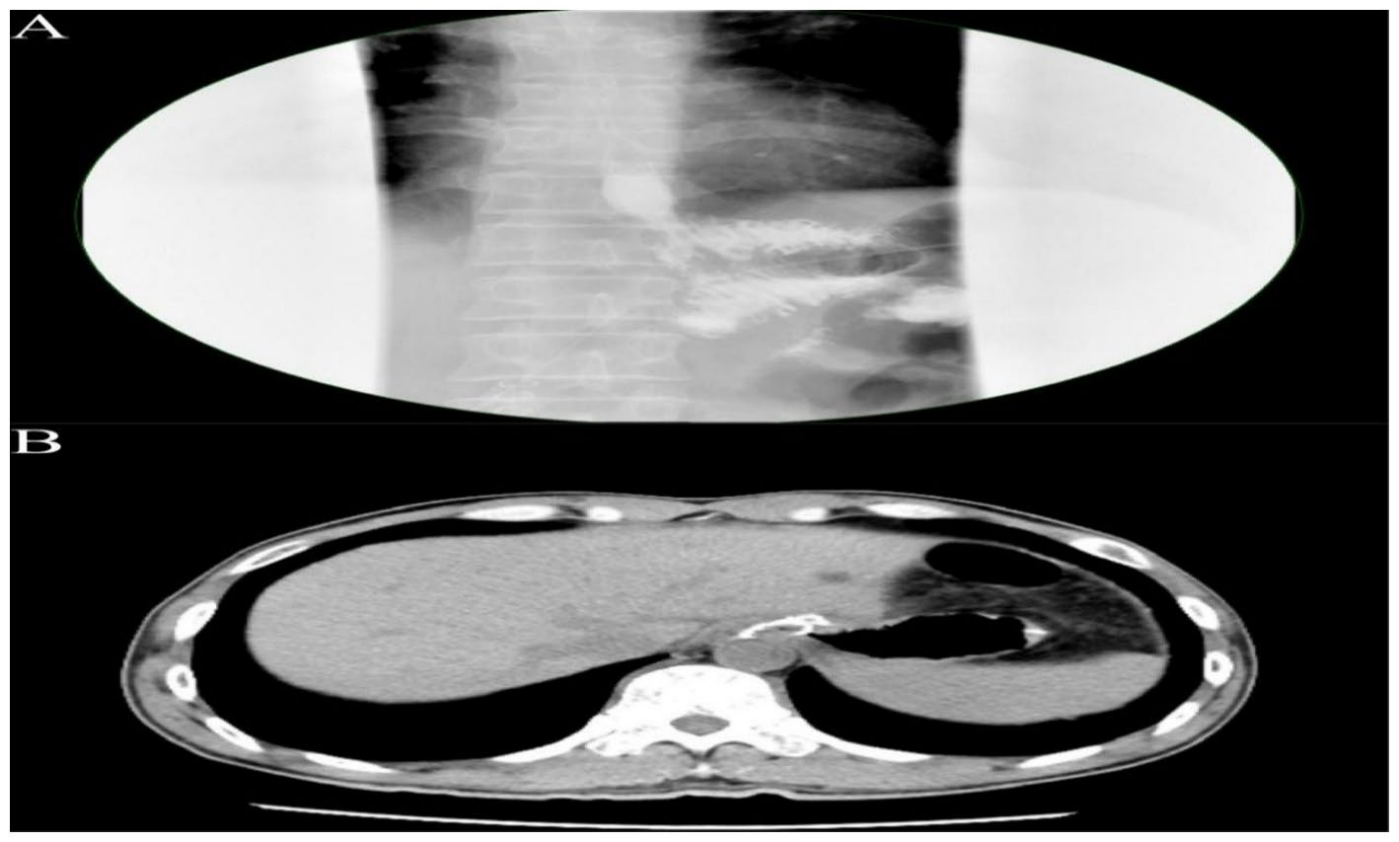
**(A)** Upper gastrointestinal contrast study demonstrating an esophagojejunostomy with an anastomotic diameter of approximately 0.6 cm. The contrast agent traverses slowly but smoothly, with no significant leakage observed around the anastomosis. **(B)** Abdominal CT scan reveals the esophagojejunostomy, showing a circumferential metallic density shadow, with the anastomosis appearing patent.

On postoperative day 3, the patient passed flatus and gradually resumed oral intake. Upper GI contrast on day 6 showed a patent anastomosis with smooth passage (Figure 4A). Sutures and the left upper abdominal drain were removed at 1 week. On day 12, abdominal CT confirmed anastomotic patency with no significant abnormalities (Figure 4B). The posterior esophagojejunostomy drain was removed, and the patient was discharged. Supportive care included albumin supplementation, nutritional support, regular dressing changes, and monitoring of liver and renal function. The patient recovered well, tolerated diet, albumin normalized, and no complications occurred. Pathology revealed marked mucosal fold hypertrophy, severe chronic active inflammation with erosion, congestion, edema, prominent foveolar hyperplasia with mucinous change, distortion, and dilation, foveolar extension to the muscularis mucosae, and reduced acid glands, consistent with Menetrier’s disease (Figure 3D). Immunohistochemistry: CEA (partial+), CK (broad-spectrum+), Ki67 (normal proliferative pattern), MUC-2(−), MUC-5 AC (foveolar+), MUC-6 (focal+), P53 (wild-type).

At 23 months post-surgery, the patient showed no hypoalbuminemia, generalized edema, or related symptoms. Body weight recovered gradually, and serum albumin remained normal.

## 3 Discussion

Hypertrophic gastropathy is a rare, acquired precancerous gastric disorder of unclear etiology (15), predominantly affecting the fundus and body (12), with occasional cases in the antrum (18). Since its first report in 1888, only several hundred cases have been documented worldwide (6). Etiology differs between children and adults; in adults, it typically occurs in males aged 30–60, is progressive, and is associated with *Helicobacter pylori* infection (13, 19). Overexpression of TGF-*α* in gastric epithelium, leading to increased EGFR signaling (20), and comorbidity with ulcerative colitis have been implicated (20, 21). SMAD4 mutations are also reported as a possible cause (10). The clinical manifestations of giant hypertrophic gastritis are often nonspecific, including epigastric pain, fatigue, anorexia, and edema (2, 3, 15, 16), necessitating differentiation from conditions such as hypertrophic lymphocytic gastritis, hypersecretory gastritis, Zollinger-Ellison syndrome, and gastric cancer (12, 19). Diagnosis typically requires comprehensive endoscopic full-thickness mucosal biopsy and laboratory tests (serum albumin, gastrin, *Helicobacter pylori*, cytomegalovirus) (12). Pathology usually reveals mucosal thickening, pit cell hyperplasia, and oxyntic gland atrophy (14). There is no standard treatment; options include non-surgical and surgical approaches. Eradication of *H. pylori* and antiviral therapy for cytomegalovirus can benefit some patients (2, 22); somatostatin analogs (23, 24) and EGFR monoclonal antibodies are effective in select cases (15, 20, 25). Other medications, such as H2R antagonists and PPIs, may alleviate symptoms (12, 26). For non-surgical patients, endoscopic monitoring every 6 months is recommended (27–29). Surgical treatment, primarily partial or total gastrectomy (via laparoscopy or robotic assistance), is considered the first-line therapy (12, 30–35). Robotic surgery is considered one of the optional approaches (35). Some cases may be cured by endoscopic submucosal dissection (ESD) (36).

We report a case of a young male with Menetrier’s disease presenting as generalized edema due to hypoproteinemia. After a complex diagnostic process, definitive diagnosis was achieved. Given poor response to medical therapy and extensive lesions, we performed robot-assisted total gastrectomy with Roux-en-Y esophagojejunostomy. Based on the patient’s clinical course, our experience with robotic treatment, and relevant literature, we summarize the following insights:

1. Giant hypertrophic gastritis presents with non-specific clinical features and a broad differential diagnosis, making definitive diagnosis challenging (16). This patient underwent seven consultations, extensive disease-related investigations, three endoscopies with biopsies, before diagnosis and optimal treatment were established, highlighting diagnostic difficulty and therapeutic uncertainty.
2. Single endoscopic biopsy may be insufficient due to inadequate depth and sampling; multiple, large, and deep biopsies from various sites may be necessary to improve diagnostic yield (15, 37). Endoscopic ultrasonography may offer diagnostic advantages (38).
3. Due to the rarity of this disease, clinicians, endoscopists, and pathologists may lack experience. Medical professionals should enhance their knowledge and consider this diagnosis in patients with extensive negative workups, pursuing targeted investigations.
4. Based on our center’s experience with the da Vinci Xi robotic system for gastrointestinal diseases, we find that patients with hypertrophic gastritis often exhibit diffuse gastric wall thickening and prominent perigastric vascular proliferation. The da Vinci robot’s precision and flexibility offer advantages in such cases (39). Careful dissection is required to avoid bleeding or vascular compromise; liver suspension aids exposure and frees the assistant. Stapler height should be selected according to gastric wall thickness to prevent misfiring or staple line disruption.
5. We used the “curved five-port” trocar layout, but due to the extensive resection range in total gastrectomy, trocar positions should be adjusted according to patient body habitus.
6. The procedure involves extensive organ mobilization and transection with complex local anatomy, requiring high technical proficiency from assistants, especially for exposure and dissection without direct vision. Team coordination is essential to enhance surgical safety.
7. Postoperative specimens showed diffuse gastric wall thickening, coarse and disorganized mucosal folds, and abundant yellow-white viscous secretions. Literature suggests that imbalance between mucus and acid secretion leads to protein malabsorption and subsequent hypoproteinemia (16).
8. As no effective treatment exists, a multidisciplinary team involving gastroenterologists, pathologists, nutritionists, oncologists, and surgeons is recommended for management (15).
9. Intraoperative complications such as bleeding or splenic injury require clear visualization and gentle manipulation; splenectomy may be necessary for uncontrolled splenic bleeding, and conversion to open surgery should be considered when needed. Early postoperative complications mainly include duodenal stump and anastomotic leaks; intraoperative assessment of tissue quality, secure suturing, adequate blood supply, and tension-free anastomosis, along with postoperative nutritional support, are essential.
10. Although surgical management of Menétrier’s disease is well-established, robotic surgery remains rare, with only one case reported in the literature (35). Although the report indicates that robotic systems could improve surgeon ergonomics and suggests potential safety and efficacy, as well as the possibility of robotics being the optimal therapeutic option, these conclusions require cautious interpretation. Due to the disease’s rarity and reliance on single-case reports, systematic comparative data on perioperative safety and long-term outcomes between robotic, laparoscopic, and open surgery are lacking. Furthermore, the relatively higher costs and potentially prolonged operative times associated with robotic surgery are critical factors that must be carefully weighed in clinical decision-making (40, 41). Although some studies indicate that workflow optimization may improve the efficiency of robotic procedures, the actual impact of these factors requires thorough evaluation (42). Demonstrating technical feasibility in isolated cases does not suffice to prove the safety of robotic platforms or their superiority to conventional methods. Future research should focus on long-term follow-up of robotic surgery patients to evaluate late outcomes and complications, and on conducting large, rigorously designed multicenter studies to systematically assess the safety, long-term efficacy, and cost-effectiveness of robotic surgery.

## 4 Conclusion

Menetrier’s disease, a rare precancerous condition, often presents with hypoalbuminemia and is easily missed. Diagnosis requires multidisciplinary collaboration, repeated deep biopsies, and imaging. While medical therapy benefits some patients, extensive disease necessitates total gastrectomy. This case highlights the need to raise awareness, standardize endoscopic biopsy, and advance minimally invasive techniques; Robot-assisted total gastrectomy is a surgical method for treating Meniere’s disease; however, its cost-effectiveness should be carefully considered, and in the long term, more high-quality studies may be required to validate its feasibility.

## Data Availability

The original contributions presented in the study are included in the article/Supplementary material, further inquiries can be directed to the corresponding author/s.
