# Supplementary figures and images for: Robotic-assisted total gastrectomy for refractory hypoproteinemia in Menetrier’s disease: a case report with operative video and literature review
Source: Front Med (Lausanne). 2025 Sep 17;12:1634451. doi: 10.3389/fmed.2025.1634451 (PMC12484214; doi:10.3389/fmed.2025.1634451)

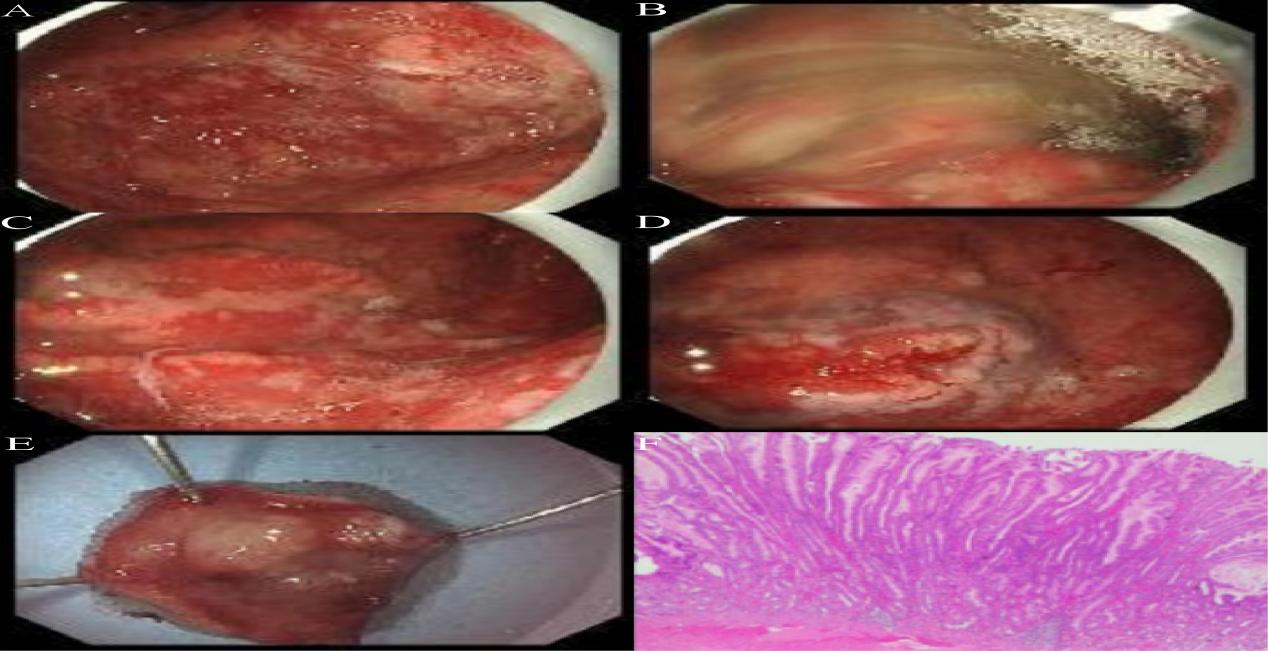

Supplement: Supplementary file 1 [file Image_1.JPEG]
